# Masking ability of layering techniques combined with white or yellow opaquers on a dark background

**DOI:** 10.4317/jced.64117

**Published:** 2026-05-29

**Authors:** Gabriela Nogueira Bouchet, Luiz Henrique Burnett-Júnior, Ana Maria Spohr

**Affiliations:** 1 Pontifical Catholic University of Rio Grande do Sul, School of Dentistry, Department of Restorative Dentistry, Porto Alegre, Brazil

## Abstract

**Background:**

Clinicians still experience hesitation and uncertainty regarding the techniques required to achieve the appropriate final color for clinical cases involving discolored teeth, and it is often unclear whether a white or yellow opaque shade should be applied. This study evaluated the influence of resin composite (RC) layering techniques, using either regular or flowable opaques in white and yellow shades, on the masking ability of a discolored substrate.

**Materials and Methods:**

Acrylic upper left central incisors, shade C3, with veneer preparations were divided into 11 groups (n=5). Shade C3 (n=5) and shade A1 (n=5) intact acrylic teeth were the negative and positive control groups, respectively. Teeth were restored with the monolayer, bilayer or trilayer technique, and white or yellow opaques were associated with dentin RC and/or enamel RC. E00 values were obtained using the CIEDE2000 formula. Data were analyzed by Kruskal-Wallis and Dunn's test (=5%).

**Results:**

Groups restored with the trilayer technique (opaque + dentin RC + enamel RC) obtained significantly lower E00 values, below the acceptability threshold. In the trilayer technique, the use of white regular opaque (1.18) did not differ significantly from the positive control (0.69) (p&gt;0.05). The use of yellow opaques increased E00, but it remained below the acceptability threshold.

**Conclusions:**

The trilayer technique using white or yellow opaques, dentin, and enamel RCs yielded E00 values below the acceptability threshold. However, white opaques were more effective in masking the discolored background.

## Introduction

One of the most common causes of dissatisfaction is tooth discoloration (1), which is often associated with dental trauma, pulp necrosis, the use of endodontic materials (2), restorative materials (3), as well as certain medications (4). In these cases, direct resin composite veneers are still popular among clinicians and their use is taught in dental schools and continuing education courses (5). This restorative technique offers several advantages, including cost-effectiveness, the possibility of process completion in a single appointment, the elimination of provisional restorations, independence from laboratory procedures (6), and satisfactory restoration longevity (7 , 8). A favorable esthetic outcome of a direct resin composite veneer results from the combination of proper contour, shape, surface texture, and color matching of the restoration compared to natural teeth (6 , 9). To achieve appropriate color matching, a wide variety of material translucencies are available, such as opaques, dentin, enamel and the body resin composite, which is considered a universal restorative that is more translucent than dentin and less translucent than enamel (10 , 11). However, the challenge of this approach is the difficulty in effectively masking dark tooth backgrounds and obtaining a natural appearance (9). In the case of a dark background, an opaquer can be used to mask a dark axial wall (9 , 12). Opaquers are light-cured dimethacrylate-based resins that contain metallic pigments, which give them a distinct hue, saturation, and opacification potential. These resin composites are described in the literature as high-opacity resins, as they provide material with greater opacifying capacity (7 , 13). When opaquers are not used, it becomes necessary to apply a thicker layer of restorative material to mask the dark background (14). Additionally, achieving this effect requires either removing additional tooth structure to deepen the preparation area or creating an overcontoured restoration (9). As an alternative, opaquers were developed to serve as the first layer of the restoration, occupying minimal physical space and allowing a more conservative and minimally invasive procedure (7 , 9 , 15 , 16). A wide range of opaquers are commercially available. These materials can be found either in regular consistency (regular opaque composites) (14) or in flowable consistency (flowable opaque composites) (17). Most commercial brands provide only one or two shade options, generally white or yellow, with the latter offered in different shades such as A1, A2 or A3. Both white (8 , 9 , 16) and yellow (7 , 13 , 15) opaques are frequently used for the treatment of tooth discoloration. In this case, a dentin-colored resin composite, which is highly chromatic and less translucent, is applied over the opaquer followed by an enamel-colored resin composite, which is more translucent (18). However, different restorative techniques can be applied, such as single-shade resin composites (19) and bilayer or trilayer techniques to explore different associations of opaquer, body, dentin, and enamel shade composites (14 , 20). A recent scoping review concluded that an acceptable masking of black backgrounds of the oral cavity is achieved by at least 1.0 mm of the opaque shade of the resin composite. Additionally, acceptable masking of a C4 shade background is achieved with either one layer of opaque shade that is at least 0.5 mm thick or by different combinations of the layering technique with a total thickness of 1.5 mm (14). Regardless, achieving optimal esthetic results requires the correct combination of colors and material thicknesses to reproduce light propagation patterns similar to those of natural dentin and enamel (18 , 21 , 22), corresponding to the acceptability and perceptibility thresholds in dentistry (23 , 24). Discolored teeth exhibit a wide range of colors and degrees of discoloration, making it unlikely that a single protocol could address every case. The thickness of the resin composite layer that is required to achieve adequate masking is variable and depends on the translucency/opacity of the tested resin composites and the background shade (14). Therefore, clinicians still experience hesitation and uncertainty regarding the necessary techniques to achieve the proper final color for each clinical case involving discolored teeth (15). With respect to opaquers, it is often unclear whether a white or yellow shade should be applied, and few studies have compared the masking ability of white and yellow opaques using the same methodology (25 , 26). Therefore, the aim of this study was to evaluate the influence of different resin composite layering techniques, using either regular or flowable opaque composites in white and yellow shades, on the masking ability of a discolored substrate. This study is based on the null hypothesis that masking ability is not affected by (i) the layering technique or (ii) the color of the opaquer.

## Materials and Methods

**Figure 1:**
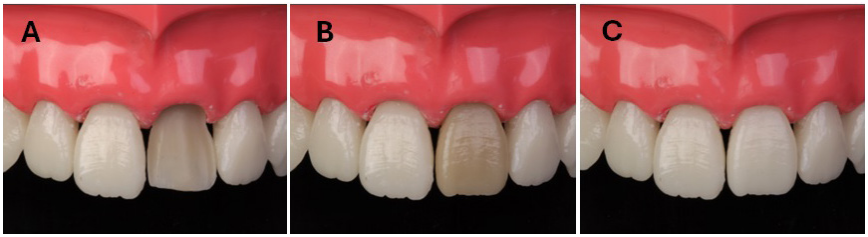
Acrylic teeth (upper left central incisors) placed in the mannequin. (A) Acrylic tooth with 2 mm deep veneer preparation, shade C3; (B) Intact acrylic tooth, shade C3 (negative control); (C) Intact acrylic tooth, shade A1 (positive control).

The study was approved by the Scientific Committee of the School of Health and Life Sciences at the Pontifical Catholic University of Rio Grande do Sul (registration no. 12093). The materials that were employed in this study are presented in Table 1, including their composition, batch number, and manufacturers. The sample size was calculated using a 95% confidence level and a margin of error of 5% of the mean, based on the standard deviation derived from the experimental data. A minimum of 5 replicates per group was estimated. The inclusion criteria consisted of upper left central incisor acrylic teeth in shade C3 (P-Oclusal Prod. Odont. Ltda, Capela do Socorro, SP, Brazil); acrylic teeth with standardized 2 mm-deep veneer preparations; specimens with intact structure, without visible defects; a standardized substrate color for the experimental groups (shade C3) and the positive control group (shade A1); and specimens with uniform dimensions and finishing to allow standardization of color measurements. The exclusion criteria were acrylic teeth with cracks or structural defects; acrylic teeth with color variations other than C3 (except for the positive control group A1); specimens with inadequate or non-standardized preparation; and irregular surfaces or defects that could compromise spectrophotometric readings. Then, a total of 65 acrylic teeth were used, representing the upper left central incisor, shade C3, with 2 mm-deep veneer preparations (Fig. 1A).

1

Acrylic teeth were randomly divided into 13 groups (n = 5) and restored with one or more of the materials listed in Table 1.

Table 1

The negative control group consisted of shade C3 intact acrylic teeth (n = 5) (Fig. 1B), whereas the positive control group consisted of shade A1 intact acrylic teeth (n = 5) (Fig. 1C). Additional acrylic teeth were restored, and the thickness of each material was determined using a caliper (Golgran, São Caetano do Sul, SP, Brazil) (Table 2).

Table 2

**Figure 2:**
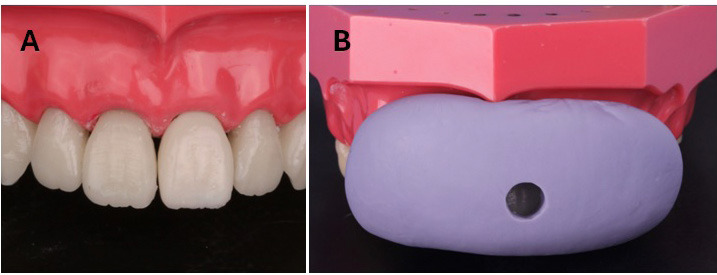
(A) Acrylic tooth restored with the trilayer technique (WRO-DE group); (B) Polyvinyl siloxane guide with the hole positioning the spectrophotometer tip on the middle third of the buccal surface.

Transparent polyvinyl siloxane guides (Silic-One Clear Body, FGM; Joinville, SC, Brazil) were fabricated to standardize the thickness of each material according to Table 2. For example, to obtain a guide number of 3, the opaquer was applied to a thickness of 0.6 mm and light-cured. Next, polyvinyl siloxane impression material was applied over the tooth, which covered the buccal and palatal surfaces, and allowed to polymerize. Thus, guides with different internal thicknesses for the material were produced as follows: guide 1 (0.2 mm), guide 2 (0.9 mm), guide 3 (0.6 mm), guide 4 (0.7 mm), and guide 5 (1.0 mm). Guide 6 corresponded to the final material layer and the thickness was adjusted to complete the restoration at a total thickness of 2 mm. The acrylic teeth were placed in a mannequin (P-Oclusal, Capela do Socorro, SP, Brazil) for the application of all material layers. A Millenium 3102 spatula (Golgran, São Caetano do Sul, SP, Brazil) was used to insert the regular opaque composites, and a #4 brush (Keramik, Vinhedo, SP, Brazil) was used for the application of flowable opaque composites. After the restorative material was applied to the tooth, the respective transparent polyvinyl siloxane guide was positioned, and digital pressure was applied to allow the excess material to flow out. Photopolymerization was conducted through a transparent guide using a VALO Grand Cordless light-curing unit (Ultradent, South Jordan, UT, USA), according to the recommendations of the manufacturer. Any excess material was removed with the aid of Sof-Lex Pop-On Discs 4931G (3M/ESPE, St. Paul, MN, USA) (Fig. 2A).

2

Restorations were polished using the Swivel Diamond Polishers 1921 CA system (Jota, Florianópolis, SC, Brazil) at 7,000-9,000 rpm under light pressure for 30 s per polishing tip with water irrigation. All procedures were performed by a single operator. A polyvinyl siloxane guide (Variotime, Kulzer, São Paulo, SP, Brazil) was used to standardize the positioning of the spectrophotometer tip on the middle third of the buccal surface (Fig. 2B). The color coordinates L*, a*, and b* of each sample were consecutively measured three times through a spectrophotometer (Easyshade Advance 4.0, VITA Zahnfabrik, Bad Sackingen, Germany) under standard lighting conditions, and mean values were obtained. Prior to the analysis, the spectrophotometer was calibrated on its charging base. Color differences were calculated using the L*, a*, and b* values of the teeth samples using the CIEDE2000 formula for each group in comparison to the positive control (shade A1) (27).


$$\mathrm{\Delta E2000}=\sqrt{{\left(\frac{\Delta L^{\prime }}{K_{L}S_{L}}\right)}^{2}+{\left(\frac{\Delta C^{\prime }}{K_{C}S_{C}}\right)}^{2}+{\left(\frac{\Delta H^{\prime }}{K_{H}S_{H}}\right)}^{2}+R_{T}\left(\frac{\Delta C^{\prime }}{K_{C}S_{C}}\right)\left(\frac{\Delta H^{\prime }}{K_{H}S_{H}}\right)}$$

where L', C', and H' are differences in luminosity (L'), chroma (C'), and hue (H'), respectively, for a pair of measurements. RT is the rotation function that accounts for the interaction between chroma and hue differences in the blue region. SL', SC', and SH are weighting functions that adjust the total E00 for variation in the location of the color difference pair in the L*, a*, and b* coordinates, and the parametric factors KL', KC' and KH are correction terms for deviation from experimental reference conditions [Luo et al., 2001]. The E00 value of the positive control group (shade A1) was calculated by comparing the specimens within the positive control group. The L* values followed a normal distribution according to the Shapiro-Wilk test, and homogeneity of variances was confirmed by Levene's test. Therefore, the L* values were analyzed using ANOVA, followed by Tukey's post hoc test. However, the E00 values did not follow a normal distribution, and heterogeneity of variances was observed. Therefore, the nonparametric Kruskal-Wallis test, followed by Dunn's post hoc test (with Holm correction) was used to analyze the E00 values. Statistical analyses were performed using SPSS software (SPSS Statistics 23.0.0; IBM, Armonk, NY, USA), and the significance level was set at 5%.

## Results

There was a significant difference among the groups for the L* and E00 values (p &lt; 0.001). The L* and E00 values for each group and the significant differences are presented in Table 3.

Table 3

With respect to L*, only the D (77.67) and E (70.21) groups and the negative control group (65.46) differed significantly from the positive control group (81.21) (p &lt; 0.05). The E00 value of WRO-DE (1.18) group was the only one that did not differ significantly from the positive control group (0.69) (p &gt; 0.05).

## Discussion

This study evaluated the ability of different restorative techniques to mask a C3 shade background, which represents severe discoloration (13 , 14), exploring both bilayer and trilayer techniques combined with white or yellow opaques as well as monolayer and bilayer techniques without the use of opaques. The difference in color (E00) was calculated between the experimental groups and the positive control group (shade A1). The E00 values are related to two distinct parameters. The perceptibility threshold (PT) refers to the lowest E00 value that can be detected by an observer and represents the limit below which the color difference is considered imperceptible. However, the acceptability threshold (AT) corresponds to the highest E00 value that is still considered esthetically tolerable and even if the color difference is perceptible (23). A study established E00 0.81 and E00 1.77 as the perceptibility and acceptability thresholds, respectively (17). Other studies reported equivalent values: E00 0.8 for perceptibility and E00 1.8 for acceptability (13 , 18), and E00 2.25 for acceptability (20). In summary, there is a consensus in the literature that E00 values less than 0.8 are considered imperceptible, whereas values up to 2.25 may be considered esthetically acceptable for composite resin restorations. In this study, all restorative groups exceeded the perceptibility threshold of E00 0.8, and only four groups obtained values that fell below the acceptability threshold of E00 2.25. Although acrylic teeth are produced in a standardized manner, the E of the control group (shade A1) was 0.69. This indicates that the spectrophotometer was able to detect a slight color difference among the five samples in the group; however, this difference would not be perceptible to the human eye. The worst E00 results were obtained for groups in which only the dentin resin composite (Group D) or the enamel resin composite (Group E) was applied at a thickness of 2 mm. The higher E00 value for Group E (10.86) than for Group D (7.73) is related to the greater translucency of the enamel resin composite, which failed to mask the darkened background (16). When the dentin and enamel resin composites in shade A1 (Group DE) were combined, each with a thickness of 1 mm, the E00 value decreased to 2.64. However, this technique was not effective at masking the darkened background, as the E00 was significantly greater than that of the control group and greater than the acceptability threshold of E00 2.25. This demonstrates the need for application of an opaquer to mask the darkened background. This study also tested bilayer and trilayer restorative techniques using opaquers. In the bilayer technique, white or yellow regular opaque composites were applied at a thickness of 1 mm followed by the application of only the dentin resin composite (1 mm) (WRO-D, YRO-D) or enamel resin composite (1 mm) (WRO-E, YRO-E). When the dentin resin composite was applied, the E00 was significantly greater than that when the enamel resin composite was applied. This might be explained by the resulting optical characteristics of the combined materials. Dentin resin composites are less translucent, and this material associated with the opaquer generated an opaque and less bright restoration. However, enamel resin composites are more translucent and this material associated with the opaquer generated a more translucent and brighter restoration (17 , 25). An increase in L* (luminance or lightness value) was also observed when the enamel resin composite was applied compared with when the dentin resin composite was applied. These differences resulted in a significantly higher E00 value for the dentin resin composite. However, all bilayer technique groups exhibited E00 values higher than the acceptability threshold. Groups that used the trilayer technique (WFO-DE, YFO-DE, WRO-DE, and YRO-DE) were the only ones that obtained acceptability values below the threshold of E00 2.25, which indicates that the restorations would be considered esthetically acceptable. Thus, the first hypothesis that the layering technique does not affect the masking ability was rejected. According to a study, the combination of opaque, dentin and enamel resin composites provides better color matching for darker substrates (17). This might be attributed to the combination of high opacity and the lightness of the first layer combined with the adequate thickness of the subsequent shaded resin composite layers (dentin and enamel) placed over the opaquer (17). With respect to the trilayer technique, the best E00 results were obtained for the WRO-DE group (E00 = 1.18), which showed the lowest E00 value and did not differ significantly from the positive control group (shade A1). In this group, a 0.6 mm thick white regular opaque composite was used, followed by the application of dentin and enamel resin composites in shade A1. However, when the white flowable opaque (WFO-DE - 1.28), yellow flowable opaque (YFO-DE - 1.85) or yellow regular opaque (YRO-DE - 1.64) layers were combined with the dentin and enamel resin composite layers, E00 values were significantly greater than those of the positive control group. Therefore, the null hypothesis that the color of the opaquer does not influence the masking ability was rejected. Although the results of this study revealed no significant differences in E00 values among the trilayer technique groups, the white opaques tended to have lower E00 values than the yellow opaques did. The same result was observed for L* (luminance or lightness value), and there was a slight tendency for higher L* for the two groups that used white opaques (WRO-DE and WFO-DE). With the white opaques, teeth became brighter (L* increases), contributing to lower E00 values. White color tends to make teeth appear less saturated (2), which influences acceptability since samples with lower saturation are more visually acceptable (24). The flowable opaque was applied with a brush at a lower thickness (0.2 mm) because of its fluid viscosity. On the other hand, regular opaque was applied with a spatula at a greater thickness (0.6 mm). However, when the flowable and regular viscosities of the white and yellow opaques were compared, the E00 values tended to be lower for the regular opaques. It is possible that the greater thickness of the regular opaque (0.6 mm) and the subsequent layer of dentin (0.7 mm) and enamel (0.7 mm) resin composites provided a better color match for the specific C3 background used in this study. These findings corroborate those of other studies that also obtained better results when a thicker opaquer was used to cover severely discolored backgrounds (7 , 21). With respect to L*, only the D, E, and negative control groups reached an L* value that differed significantly from that of the positive control group. These findings reinforce that the use of an opaquer is important for restoring the luminosity of the darkened tooth (shade C3) closer to that of the positive control tooth (shade A1). The YRO-E group had the closest L* value to that of the positive control group. However, the YRO-E group had a E00 value above the acceptability threshold (E00 = 3.31). This finding indicates that lightness is not the most important factor involved in determining color differences but rather that the final color of the restoration is a result of combined optical properties of all the layers (7 , 14). This is an interesting observation because when a tooth is described as "darkened," it is generally understood that the main issue to address is the darkening itself. This highlights that E00 is the result of a combination of factors; each case must be evaluated individually, and no protocol can be considered absolute. Under clinical conditions, restorations may appear to match well immediately after placement when the surface is dry, but when the surface becomes wet, changes in translucency can cause noticeable color differences (28). Although this study was conducted under laboratory conditions, the influence of surface moisture on color and translucency was not evaluated, and this factor may produce differences between laboratory results and clinical situations. Another limitation of this study are related to the use of acrylic teeth to simulate a darkened substrate, a material that exhibits different characteristics from natural teeth and does not accurately replicate the optical properties of natural dental tissues, particularly translucency and light transmission. Additionally, only a single discolored background and four opaquers associated with one brand of resin composite were used to assess the ability to mask discoloration. In clinical practice, tooth discoloration varies widely in both type and severity. Therefore, the E00 values obtained in this study should be extrapolated to clinical conditions with caution, and the findings cannot be generalized to a broader range of clinical situations. Future research should be conducted using natural teeth and a wider variety of opaquers and resin composites.

## Conclusions

The following conclusions were drawn based on the findings of this in vitro study: - The trilayer technique using white or yellow opaque, dentin, and enamel resin composites yielded E00 values below the acceptability threshold; thus, this technique was considered esthetically acceptable. - The trilayer technique using white opaque, dentin, and enamel resin composites yielded the lowest difference in color (E00) between the restored specimens and the A1 reference. - Additionally, none of the restorative techniques obtained E00 values below the perceptibility threshold.

## Figures and Tables

**Table 1:** Materials used in this study.

Material	Composition	Type	Batch number	Manufacturer
Final Touch White	Inorganic filler in a methacrylate matrix	White opaque, flowable viscosity	2148569	Voco, Cuxhaven, Germany
Opaque Empress Direct	Dimethacrylates, barium glass, ytterbium trifluoride, Ba–Al fluoro-silicate glass, and mixed spheroidal oxides, catalysts, stabilizers, and pigments	Yellow opaque, flowable viscosity	Z05YLT	Ivoclar-Vivadent, Schaan, Liechtenstein
Filtek Dentin Z350 XT WD	Bis-GMA, TEGDMA, bisphenol A polyethylene glycol diether dimethacrylate, UDMA, silane-treated ceramic, silane-treated silica	White opaque, regular viscosity	2405900225	3M/ESPE, St. Paul, MN, USA
Opaquer Forma	Bis-GMA, TEGDMA, Bis-EMA, and UDMA,zirconia/silica and barium glass	Yellow opaque, regular viscosity	D0OW2	Ultradent, Indaiatuba, SP, Brazil
Forma Resin DA1	Bis-GMA, TEGDMA, Bis-EMA, and UDMA,zirconia/silica and barium glass	Resin composite for dentin, shade A1	D0N5A	Ultradent, Indaiatuba, SP, Brazil
Forma Resin EA1	Bis-GMA, TEGDMA, Bis-EMA, and UDMA,zirconia/silica and barium glas	Resin composite for enamel, shade A1	D0P15	Ultradent, Indaiatuba, SP, Brazil

Bis-GMA: Bisphenol A glycidyl methacrylate; Bis-EMA: Ethoxylated bisphenol A dimethacrylate; TEGDMA: triethylene glycol dimethacrylate; UDMA: Urethane dimethacrylate

**Table 2:** Description of the groups in relation to the thicknesses of each material and the silicone guides used for each step.

Groups	Layers	Material	Thickness
D	Single layer	Dentin resin composite A1 (D)	2.0 mm
E	Single layer	Enamel resin composite A1 (E)	2.0 mm
DE	Layer 1	Dentin resin composite A1 (D)	1.0 mm
	Layer 2	Enamel resin composite A1 (E)	1.0 mm
WRO-D	Layer 1	White regular opaque (WRO)	1.0 mm
	Layer 2	Dentin resin composite A1 (D)	1.0 mm
YRO-D	Layer 1	Yellow regular opaque (YRO)	1.0 mm
	Layer 2	Dentin resin composite A1 (D)	1.0 mm
WRO-E	Layer 1	White regular opaque (WRO)	1.0 mm
	Layer 2	Enamel resin composite A1 (E)	1.0 mm
YRO-E	Layer 1	Yellow regular opaque (YRO)	1.0 mm
	Layer 2	Enamel resin composite A1 (E)	1.0 mm
WRO-DE	Layer 1	White regular opaque (WRO)	0.6 mm
	Layer 2	Dentin resin composite A1 (D)	0.7 mm
	Layer 3	Enamel resin composite A1 (E)	0.7 mm
WFO-DE	Layer 1	White flowable opaque (WFO)	0.2 mm
	Layer 2	Dentin resin composite A1 (D)	0.9 mm
	Layer 3	Enamel resin composite A1 (E)	0.9 mm
YRO-DE	Layer 1	Yellow regular opaque (YRO)	0.6 mm
	Layer 2	Dentin resin composite A1 (D)	0.7 mm
	Layer 3	Enamel resin composite A1 (E)	0.7 mm
YFO-DE	Layer 1	Yellow flowable opaque (YFO)	0.2 mm
	Layer 2	Dentin resin composite A1 (D)	0.9 mm
	Layer 3	Enamel resin composite A1 (E)	0.9 mm

2

**Table 3:** Mean L’ values obtained with the spectrophotometer and ∆E00 values obtained using the CIEDE2000 formula.

Groups	L*	∆E00
Negative control (C3)	65.46 F (±0.33)	12.21 A (±0.24)
D	77.67 D (±1.35)	7.73 C (±0.73)
E	70.21 E (±0.66)	10.86 AB (±0.66)
DE	79.97 BCD (±0.55)	2.64 EF (±0.49)
WRO-D	80.03 BCD (±2.16)	6.78 CD (±0.96)
YRO-D	78.99 CD (±1.20)	8.54 BC (±1.19)
WRO-E	82.8 A (±1.45)	4.59 DE (±0.61)
YRO-E	81.41 ABCD (±0.80)	3.31 E (±1.00)
WRO-DE	82.3 AB (±0.34)	1.18 GH (±0.38)
WFO-DE	82.62 AB (±1.45)	1.28 G (±0.44)
YRO-DE	81.47 AB (±0.38)	1.64 G (±0.42)
YFO-DE	81.48 AB (±1.29)	1.85 FG (±0.48)
Positive control (A1)	81.21 ABC (±0.21)	0.69 H (±0.35)

Values of L* followed by different letters in the columns differ significantly from each other according to Tukey’s test (p < 0.05).Values of ΔE00 followed by different letters in the columns differ significantly from each other according to Dunn’s test (p < 0.05).
